# Causal association of type 2 diabetes with central retinal artery occlusion: a Mendelian randomization study

**DOI:** 10.3389/fendo.2024.1379549

**Published:** 2024-08-08

**Authors:** Tong Liu, Qingli Lu, Zhongzhong Liu, Xuemei Lin, Linna Peng, Xiping Lu, Weiyan Guo, Pei Liu, Na Zhang, Songdi Wu

**Affiliations:** ^1^ Department of Neurology & Neuro-ophthalmology, The First Hospital of Xi’an (The First Affiliated Hospital of Northwestern University), Xi’an, China; ^2^ Xi’an Key Laboratory for Innovation and Translation of Neuroimmunological Diseases, Xi’an, China

**Keywords:** central retinal artery occlusion, type 2 diabetes, Mendelian randomization, causal relationship, risk factor

## Abstract

**Background:**

Central retinal artery occlusion (CRAO) is a medical condition characterized by sudden blockage of the central retinal artery, which leads to a significant and often irreversible loss of vision. Observational studies have indicated that diabetes mellitus is a risk factor for CRAO; however, there is no research on the causal relationship between diabetes mellitus, particularly type 2 diabetes, and CRAO. This study aimed to perform Mendelian randomization (MR) analysis to clarify the causal relationship between type 2 diabetes and CRAO.

**Methods:**

Genetic variants associated with type 2 diabetes were selected from two different datasets. A recent genome-wide association study of CRAO conducted using the FinnGen database was used as the outcome data. A two-sample MR was performed to evaluate the causal relationship between type 2 diabetes and CRAO. Inverse variance weighting was the primary method, and MR-Egger, maximum likelihood, and median weighting were used as complementary methods. A multivariate MR (MVMR) analysis was performed to further evaluate the robustness of the results. Cochran’s Q test, MR-Egger intercept test, and MR-PRESSO global test were used for the sensitivity analyses.

**Results:**

Genetically predicted type 2 diabetes was causally associated with CRAO(odds ratio [OR] =2.108, 95% confidence interval [CI]: 1.221–3.638, P=7.423×10^-3^), which was consistent with the results from the validation dataset (OR=1.398, 95%CI: 1.015–1.925, P=0.040). The MVMR analysis suggested that type 2 diabetes may be an independent risk factor for CRAO (adjusted OR=1.696; 95%CI=1.150–2.500; P=7.655×10^-3^), which was assumed by the validation dataset (adjusted OR=1.356; 95%CI=1.015–1.812; P=0.039).

**Conclusion:**

Our results show that genetically predicted type 2 diabetes may be causally associated with CRAO in European populations. This suggests that preventing and controlling type 2 diabetes may reduce the risk of CRAO.

## 1 Introduction

Central retinal artery occlusion (CRAO) is caused by the obstruction of the central retinal artery by thromboembolism or vasospasm, with or without retinal ischemia (1). The incidence of CRAO is estimated at 1–2/100,000 people per year (2). Clinically, CRAO is typically characterized by a painless and sudden loss of vision (3). Despite over 150 years of research involving intravenous or intra-arterial thrombolysis and conservative treatments such as regulating intraocular pressure or vasodilating retinal vasculature, an optimal management plan for CRAO has not been clearly defined (1).

Previous studies have indicated that CRAO is associated with ipsilateral internal carotid artery stenosis and various cardiovascular risk factors such as obesity, hypertension, tobacco usage, hypercholesterolemia, and diabetes mellitus (1, 4–6). A study by Dziedzic et al. revealed that 81.7% of patients with CRAO were affected by obesity or were overweight as an atherosclerotic risk factor (4). Furthermore, a retrospective analysis of patients with non-inflammatory ocular vascular conditions found that 79.8% of those with CRAO had hypertension (5). In a Polish single-center case-control study, the CRAO group displayed significantly higher risk factors such as hypertension, diabetes, hypercholesterolemia, and smoking compared to the control group (6).

Type 2 diabetes, also known as non-insulin-dependent diabetes mellitus, is a chronic metabolic disorder characterized by hyperglycemia (7). Several studies have examined the clinical characteristics and outcomes of diabetes mellitus in patients with CRAO. Sawada et al. indicated diabetes mellitus as an etiological factor of CRAO in young patients (8). Further, research by Glueck et al. and the European Assessment Group for Lysis in the Eye Study reported diabetes mellitus prevalence rates among CRAO patients of 16% and 14%, respectively (9, 10). Studies on retinal artery occlusion (RAO) patients in the United States indicate that nearly one-quarter of these patients suffer from diabetes (11, 12).

Research on the relationship between type 2 diabetes and CRAO is limited, predominantly comprising observational studies that may have been influenced by confounding factors. To date, no research has investigated the causal relationship between type 2 diabetes and CRAO at the genetic level. Mendelian randomization (MR) is a reliable method for estimating causal relationships and is less susceptible to conventional confounding issues (13). Our study aimed to elucidate the causal relationship between genetic predisposition to type 2 diabetes and CRAO using MR analysis.

## 2 Methods

### 2.1 Study design

The Strengthening the Reporting of Observational Studies in Epidemiology using Mendelian Randomization guidelines were followed in this MR study (13).

**Figure 1:**
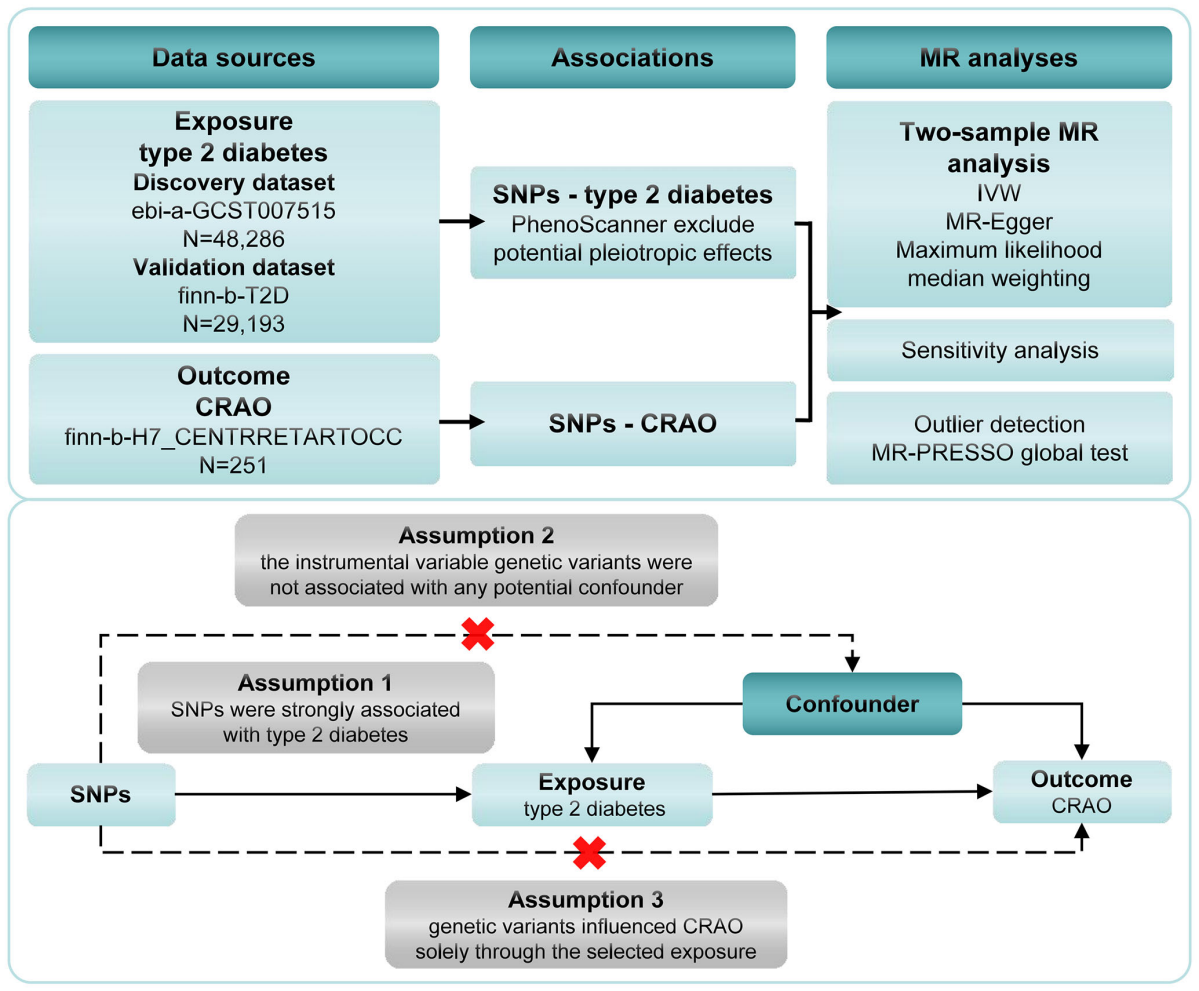
Framework for the Mendelian randomization analysis of type 2 diabetes and CRAO. CRAO, central retinal artery occlusion; SNPs, instrument single nucleotide polymorphisms; IVW, inverse variance weighting.

Two-sample magnetic resonance imaging MR and multivariate MR (MVMR) were performed to evaluate the causal relationship between type 2 diabetes and CRAO. Summary statistics were obtained from a genome-wide association study (GWAS). The foundational assumptions for our MR inference included as follows: Assumption 1, that instrument single nucleotide polymorphisms (SNPs) were strongly associated with type 2 diabetes (P<5.0×10^-8^); assumption 2, the instrumental variable genetic variants were not associated with any potential confounders; and assumption 3, genetic variants influenced CRAO solely through the selected exposure. A brief overview of the research process and foundational assumptions for our MR study is presented in **Figure 1**. While our study utilized publicly available data that did not require formal ethical approval, we recognize the significant importance of potential risks related to data privacy in genetic research. Since the data are anonymized and publicly accessible, they do not directly involve or impact individual participants. Nevertheless, we maintained rigorous ethical standards in data handling, analysis, and reporting to uphold integrity and privacy respect in studies using public database information.

### 2.2 Selection of data sources

Genetic variants associated with type 2 diabetes were selected from two different datasets. The discovery dataset, pulled from a meta-analysis conducted by Mahajan et al. (14), encompasses SNPs from 48,286 patients and 250,671 controls, all of European ancestry. Similarly, the validation dataset, also based on patients of European ancestry, includes SNPs from 29,193 cases and 182,573 controls. We based the outcome data, on a recent GWAS on CRAO employing the FinnGen database, which comprised 251 cases and 203,018 control participants, all of European ancestry. Due to the low incidence rate of CRAO, sample size estimation was not conducted using conventional methods (2). **Table 1** presents the datasets employed for these summary statistics.

**Table 1:** Characteristics of the GWASs used in the Mendelian randomization.

Traits	Population	GWAS datas	Sample sizes	No. of SNPs
Type 2 diabetes (Discovery)	European	ebi-a-GCST007515	48,286 cases and 250,671 controls	190,486
Type 2 diabetes (Validation)	European	finn-b-T2D	29,193 cases and 182,573 controls	16,380,433
hypertension	European	finn-b-I9_HYPTENS	55,917 cases and 162,837 controls	16,380,466
Obesity	European	finn-b-E4_OBESITY	8,908 cases and 209,827 controls	16,380,465
HDL-C	European	met-d-HDL_C	115,078 Sample	12,321,875
LDL-C	European	met-d-LDL_C	115,078 Sample	12,321,875
triglycerides	European	met-d-Total_TG	115,078 Sample	12,321,875
Current tobacco smoking	European	ukb-b-223	462,434 Sample	9,851,867
Type 1 diabetes	European	ebi-a-GCST010681	24,840 Sample	12,783,129
CRAO	European	finn-b-H7_CENTRRETARTOCC	251 cases and 203,018 controls	16,380,385

GWAS, genome-wide association study; SNP, single nucleotide polymorphism; HDL-C, high-density lipoprotein cholesterol; LDL-C, low-density lipoprotein cholesterol; CRAO, Central retinal artery occlusion.

We selected SNPs (P<5.0×10^−8^, linkage disequilibrium R^2^ < 0.001, clumping window = 10,000), discarded palindromic SNPs, and obtained the primary instrumental variables of type 2 diabetes. Reasons for threshold choices included ensuring that only SNPs with the strongest statistical evidence of association with type 2 diabetes were included, thus minimizing false positives and enhancing result reliability (15). This approach also aimed to reduce the risk of confounding due to neighboring SNPs and improve the validity of causal inference. Additionally, the thresholds were set to reduce redundancy and potential biases from closely linked variants, ultimately providing a more representative set of genetic instruments for studying type 2 diabetes (16).

Each primary instrumental variable was searched using PhenoScanner V2 to identify potential confounders. Any instrumental variable found to be associated with these potential confounders, or those that were not researched, were deleted to exclude potential pleiotropic effects (17). Hypertension, obesity, high-density lipoprotein cholesterol (HDL-C), low-density lipoprotein cholesterol (LDL-C), triglycerides, and current tobacco smoking have been identified as potential confounders (1). We also removed SNPs associated with type 1 diabetes to enhance rigor. Although research has demonstrated a heightened risk of diabetes mellitus associated with CRAO, distinctions between type 1 and type 2 diabetes have not been elucidated. Conversely, type 1 diabetes is linked with significantly elevated risks of stroke, likely related to CRAO (1, 18). F-statistics were utilized to evaluate the robustness of the instrumental variables for the analyzed SNPs (19).

### 2.3 Statistical analysis

We ensured that the effect alleles were harmonized across the type 2 diabetes and CRAO datasets. In the two-sample MR analysis, the inverse variance weighting (IVW) method was the main analytical method used to assess the potential pleiotropy of instrument SNPs. MR-Egger, maximum likelihood, and median weighting were used to complement the IVW estimate (13). The MR-PRESSO global test was used to identify and remove outliers (20). Statistical power was calculated with a two-sided significance test (https://shiny.cnsgenomics.com/mRnd/, α=0.05) (21).

In order to enhance the robustness of the results, we utilized MVMR to adjust for potential confounding variables, including hypertension, obesity, HDL-C, LDL-C, triglycerides, current tobacco smoking, and type 1 diabetes (1, 18).

The analysis was conducted using Package TwoSampleMR (version 0.5.7), PRESSO (version 1.0), and MVMR (version 0.4), all of which were executed within the R software environment (version 4.3.1).

### 2.4 Sensitivity Analyses

Various methods were used for the sensitivity analyses. Cochran’s Q test was utilized to assess the heterogeneity (22). The MR-Egger intercept test was used to evaluate the average directional pleiotropy, and the MR-PRESSO global test was used to adjust for the horizontal pleiotropy (23). Leave-one-out sensitivity analyses was used to detect single SNPs that caused disproportionate. Forest and funnel plots were generated to illustrate pleiotropy directly.

## 3 Results

We identified 67 SNPs from the discovery dataset (ebi-a-GCST007515) and 55 SNPs from the validation dataset (finn-b-T2D) as primary instrumental variables (**Supplementary Datasets 1**, **2**). After excluding potential pleiotropic effects (**Supplementary Datasets 3**, **4**), 38 SNPs were screened in the discovery dataset, and the variances ranged from 0.041% to 0.466%. All the F-statistics exceeded the empirical threshold of 10, suggesting that the presence of weak instrument bias was highly unlikely. This indicates that the study has a high capacity to detect real effects. In the validation dataset, the variance explained by the 39 SNPs ranged from 0.174% to 3.005%, and the F-statistics were all greater than 10. The screened SNPs and F-statistics for each dataset are presented in **Tables 2**, **3**.

**Table 2:** The screened SNPs and F statistics in the dataset of ebi-a-GCST007515.

No.	SNP	R^2^	F	No.	SNP	R^2^	F
1	rs340874	0.001031	308.40	20	rs12571751	0.001455	435.52
2	rs7572857	0.000716	214.16	21	rs5015480	0.002467	739.45
3	rs243021	0.001042	311.96	22	rs2237895	0.002773	831.31
4	rs1801282	0.001439	430.91	23	rs11603334	0.001304	390.26
5	rs11708067	0.001721	515.42	24	rs10830963	0.002937	880.62
6	rs4607103	0.00062	185.38	25	rs10842994	0.00116	347.13
7	rs1801212	0.001758	526.54	26	rs1531343	0.000953	285.07
8	rs6813195	0.001007	301.25	27	rs3764002	0.000412	123.11
9	rs35658696	0.001345	402.67	28	rs55834942	0.000944	282.38
10	rs4457053	0.000637	190.66	29	rs1359790	0.001379	412.84
11	rs9379084	0.001293	387.14	30	rs7177055	0.001308	391.69
12	rs864745	0.002769	830.20	31	rs4502156	0.000646	193.40
13	rs2191349	0.001179	352.86	32	rs8042680	0.000947	283.25
14	rs730497	0.000616	184.39	33	rs7501939	0.001732	518.80
15	rs516946	0.00148	442.99	34	rs781831	0.000832	248.95
16	rs10965250	0.004662	1400.13	35	rs8108269	0.001388	415.39
17	rs2796441	0.000695	208.01	36	rs4812831	0.000865	258.84
18	rs60980157	0.001356	405.85	37	rs738409	0.00057	170.53
19	rs10906115	0.000557	166.55	38	rs41278853	0.000933	279.10

SNP, single nucleotide polymorphism.

**Table 3:** The screened SNPs and F statistics in the dataset of finn-b-T2D.

No.	SNP	R^2^	F	No.	SNP	R^2^	F
1	rs62137406	0.001811	384.20	21	rs10882099	0.003106	659.80
2	rs112694524	0.002119	449.70	22	rs182788819	0.001795	380.88
3	rs6786846	0.002347	498.21	23	rs73541184	0.003186	676.74
4	rs11712037	0.00344	730.95	24	rs2237897	0.005844	1244.93
5	rs71330995	0.002576	547.01	25	rs78470967	0.004496	956.49
6	rs6780171	0.003731	793.06	26	rs73113806	0.002958	628.32
7	rs3887925	0.001832	388.69	27	rs112108223	0.006091	1297.74
8	rs1046317	0.0033	701.07	28	rs76895963	0.015618	3359.70
9	rs76177300	0.002052	435.42	29	rs56348580	0.002481	526.73
10	rs2781655	0.001989	421.97	30	rs7998259	0.002798	594.13
11	rs9505086	0.002035	431.77	31	rs12449219	0.001879	398.69
12	rs878521	0.002638	560.01	32	rs55993634	0.004269	907.89
13	rs62492368	0.002668	566.55	33	rs11263763	0.002047	434.37
14	rs77655131	0.002936	623.50	34	rs7224685	0.001736	368.33
15	rs10245867	0.001993	422.98	35	rs2303700	0.001984	421.01
16	rs11558471	0.003133	665.48	36	rs7507893	0.00186	394.70
17	rs28642213	0.004325	919.86	37	rs8100204	0.002494	529.49
18	rs10965246	0.004093	870.25	38	rs45551238	0.004922	1047.55
19	rs34872471	0.030048	6560.11	39	rs8353	0.002335	495.65
20	rs11257658	0.002879	611.43				

SNP, single nucleotide polymorphism.

Evaluation of the association between type 2 diabetes and CRAO risk is shown in **Table 4**. The results of the IVW method indicated that type 2 diabetes was causally associated with a heightened risk of CRAO (odds ratio [OR] =2.108, 95% confidence interval [CI]:1.221–3.638, P=7.423×10^-3^). This effect was supported by the maximum likelihood (OR=2.130, 95%CI:1.227–3.695, P=7.176×10^-3^) and weighted median (OR=2.394, 95%CI:1.095–5.235, P=2.871×10^-2^) methods. The validation dataset confirming the causal effect of type 2 diabetes yielded consistent conclusions. Moreover, the MR-PRESSO global test did not detect outliers in any of the instrumental variables used.

**Table 4:** The evaluation of the association between type 2 diabetes and CRAO.

Traits	N	Method	OR	95% CI	P
ebi-a-GCST007515	38	Inverse variance weighted	2.108	1.221–3.638	7.423×10^-3^
		MR Egger	3.052	0.499–18.682	0.235
		Maximum likelihood	2.130	1.227–3.695	7.176×10^-3^
		Weighted median	2.394	1.095–5.235	2.871×10^-2^
finn-b-T2D	39	Inverse variance weighted	1.398	1.015–1.925	0.040
		MR Egger	1.365	0.728–2.559	0.339
		Maximum likelihood	1.400	1.015–1.932	0.041
		Weighted median	1.429	0.861–2.371	0.167

MR, mendelian randomization; OR, odds ratio; CI, confidence interval.

**Figure 2:**
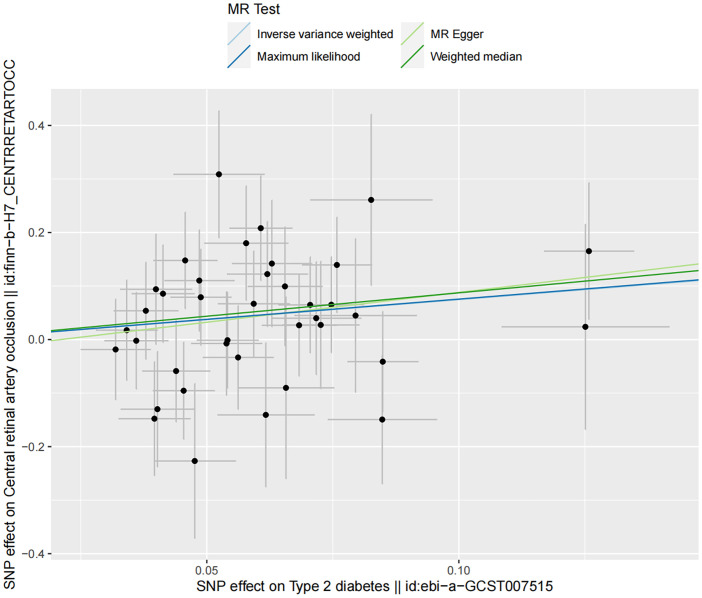
Scatter plot illustrating the risk of CRAO associated with type 2 diabetes, derived from the dataset ebi-a-GCST007515, analyzed using IVW, MR-Egger, Maximum Likelihood, and Median Weighting methods.

**Figure 3:**
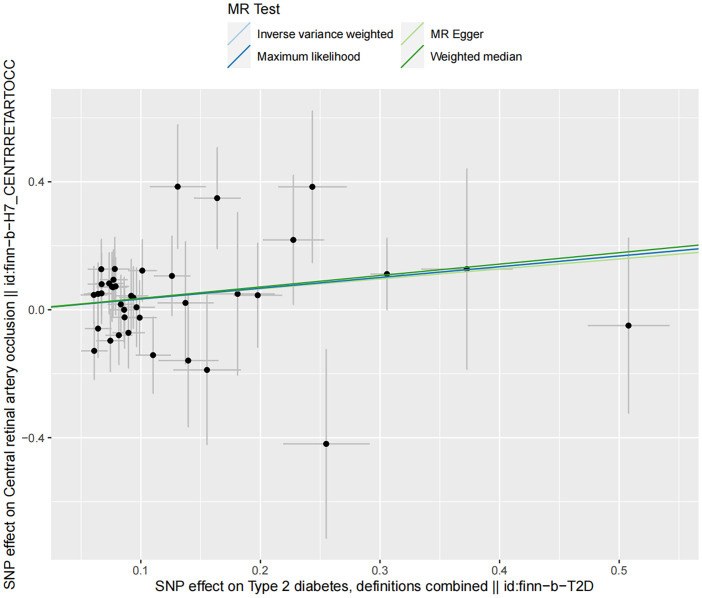
Scatter plot illustrating the risk of CRAO associated with type 2 diabetes, derived from the dataset finn-b-T2D, analyzed using IVW, MR-Egger, Maximum Likelihood, and Median Weighting methods.

Sensitivity analyses demonstrated no presence of heterogeneity and horizontal pleiotropy (**Table 5**). In the discovery dataset, the Cochran’s Q test derived P was 0.474 for IVW and 0.435 for MR-Egger, indicating no heterogeneity. The MR-Egger intercept test showed no evidence of a significant intercept (intercept=-0.023, P=0.677). In addition, the MR-PRESSO global test did not identify any evidence of horizontal pleiotropy (P=0.485). Moreover, sensitivity analyses of the validation dataset was consistent with those of the discovery dataset. The scatter plots depicted the causal relationship between type 2 diabetes and CRAO (**Figures 2**, **3**). Leave-one-out analysis indicated that no individual genetic variant had strong influence on the association between type 2 diabetes and CRAO (**Supplementary Figures**).

**Table 5:** Test for heterogeneity and horizontal pleiotropy.

Two-Sample MR analysis	SNPs	Heterogeneity		Horizontal pleiotropy		
		P for IVW	P for MR-Egger	MR-PRESSO global test P-value	MR-Egger intercept	Intercept P-value
ebi-a-GCST007515	38	0.474	0.435	0.485	-0.023	0.677
finn-b-T2D	39	0.819	0.785	0.839	0.003	0.931

SNP, single nucleotide polymorphism; MR, mendelian randomization; IVW, inverse variance weighting.

For the discovery dataset, the calculated power value was 98%, indicating a strong ability to detect real effects and determine whether type 2 diabetes acted as a risk factor for CRAO. In the validation dataset, the obtained power value of 70% somewhat supported the findings.

The results of the MVMR analysis showed that type 2 diabetes was associated with a significantly increased risk of CRAO (**Table 6**).

**Table 6:** MVMR results.

Adjustment for confounding factors	type 2 diabetes dataset	Number of SNPs	CRAO dataset	OR	95%CI	P
Hypertension, obesity, HDL-C, LDL-C, triglycerides, current tobacco smoking and Type 1 diabetes	ebi-a-GCST007515	44	finn-b-H7_CENTRRETARTOCC	1.696	1.150–2.500	7.655×10^-3^
Hypertension, obesity, HDL-C, LDL-C, triglycerides, current tobacco smoking and Type 1 diabetes	finn-b-T2D	30	finn-b-H7_CENTRRETARTOCC	1.356	1.015–1.812	3.935×10^-2^

CRAO, Central retinal artery occlusion; HDL-C, high-density lipoprotein cholesterol; LDL-C, low-density lipoprotein cholesterol; SNP, single nucleotide polymorphism; OR, odds ratio; CI, confidence interval.

## 4 Discussion

MR analysis has been a popular method for establishing causal relationships and has unearthed new perspectives into disease mechanisms. Our study evaluated and suggested the causal relationship between type 2 diabetes and CRAO using patient data based on European ancestry with a two-sample MR.

So far, most studies have focused on the risk of diabetes mellitus on RAO. Furthermore, an analysis of the English national linked Hospital Episode Statistics indicated a significant association between diabetes mellitus and an increased risk of RAO (rate ratio=9.31, 95%CI: 8.26–10.50) based on the population of England (24). Additionally, Calway et al. investigated potential risk factors for perioperative RAO in cardiac surgery from the United States National Inpatient Sample. They observed that diabetes mellitus with ophthalmic complications was a risk factor for RAO during cardiac surgery, while type 2 diabetes was associated with a lower risk (25). However, these studies did not specifically explore RAO subtypes related to diabetes mellitus.

The association between CRAO and diabetes mellitus has rarely been investigated in population-based studies. Hayreh et al. classified RAO into CRAO and branch artery occlusion in a cohort study, demonstrating a significantly higher prevalence of diabetes mellitus compared to that in a matched US population (P< 0.0001) (12, 18). A cohort study encompassing 482,392 participants (mean age, 55.06 years), indicated a 2.28-fold increased risk of CRAO in Taiwanese patients with diabetes mellitus (188 patients with CRAO and diabetes mellitus, 86 patients in the control group) (26). According to a Scientific Statement From the American Heart Association, the incidence of CRAO is heightened with the presence of vascular risk factors such as hypertension, hyperlipidemia, diabetes mellitus, tobacco exposure, and obesity, as supported by some studies (1). Notably, the current study did not differentiate between diabetes type 1 and 2 in its analysis. Moreover, most risk factors were not consistently adjusted for in the epidemiological data.

Our two-sample MR analysis using the IVW method suggested a significant causal association between type 2 diabetes and CRAO (discovery dataset: OR=2.108, 95%CI: 1.221–3.638, P=7.423×10^-3^; validation dataset: OR=1.398, 95%CI: 1.015–1.925, P=0.040). This association was confirmed using sensitivity analyses. SNPs associated with potential confounders were removed to reveal the direct effects of type 2 diabetes on CRAO. MVMR analysis suggested that type 2 diabetes was an independent risk factor for CRAO (ebi-a-GCST007515: adjusted OR=1.696, 95%CI=1.150–2.500, P=7.655×10^-3^; finn-b-T2D: adjusted OR=1.356, 95%CI=1.015–1.812, P=0.039).

Although the biological mechanisms through which diabetes mellitus increases the susceptibility to CRAO remain unclear, several possible mechanisms have been proposed (27). Firstly, the most common cause of CRAO is the obstruction of the central retinal artery due to emboli that originate from the ipsilateral internal carotid artery, aortic arch, or heart (1). Individuals diagnosed with type 2 diabetes show a higher presence of lipid-rich necrotic core (LRNC). In these patients, carotid plaques containing LRNC% > 22.0% are defined as a risk factor for the emergence of acute cerebral infarction lesions restricted to the carotid region (28). Secondly, from a gene-specific perspective, atrial fibrillation patients carrying clonal haematopoiesis of indeterminate potential (CHIP) gene mutations have a higher prevalence of diabetes, and they are also at a higher risk of developing composite clinical events, including ischemic stroke (29). This highlights the genetic connections influencing both cardiovascular and ocular health in diabetic patients. Thirdly, concerning inflammatory, diseases like giant cell arteritis (GCA) that affect the proximal ophthalmic artery can induce simultaneous ischemia of the retina and the optic nerve head (1, 30). Diabetes increases the likelihood of developing GCA, and the subsequent necessity for glucocorticoid therapy in these patients can further elevate the risk of diabetes onset (31–33). Diabetes mellitus also impacts the ocular inflammatory mediators and immune-competent cells (32, 34). Dysregulation of the innate immune system is associated with the occurrence and progression of GCA (35). In cases of poorly controlled diabetes, certain immune responses may lead to dysbiosis of the ocular surface microbiome and an increase in harmful bacteria (32, 36). Additionally, alterations in the gut microbiome of patients with type 2 diabetes could potentially increase the risk of CRAO, suggesting a novel area for further investigation into the systemic conditions affecting ocular health (37).

The incidence of CRAO in patients with diabetes mellitus calls for an urgent, interdisciplinary approach, underscoring the necessity for tight collaboration between neuro-ophthalmologists and endocrinologists. Neuro-ophthalmologists are integral in addressing acute retinal ischemia and enhancing visual outcomes. Their responsibilities include conducting prompt ophthalmological examinations, comprehensive neurological evaluations, and brain computed tomography scans; orchestrating diagnostic procedures; recommending treatments, such as intravenous tissue plasminogen activator (tPA), intra-arterial tPA, or conservative methods; and meticulously monitoring the patient’s condition during treatment (1, 38). Endocrinologists, aware of the severe visual complications associated with CRAO, should offer pertinent health advice to patients with diabetes mellitus, encouraging emergency referrals and advocating for early preventive interventions. Strengthened collaboration between endocrinologists and neuro-ophthalmologists is pivotal in diminishing the risk of CRAO and lessening visual impairment in patients with diabetes mellitus.

Our study possesses several strengths. First, an association between genetic susceptibility to type 2 diabetes and CRAO by MR analysis is reported for the first time. Second, genetic variants associated with type 2 diabetes were selected from two different datasets, and consistent results were obtained. Third, to avoid traditional confounding factors and inverse causality, MVMR was performed to confirm the results of the two-sample MR.

However, our study has some limitations. First, MR analysis using genetic variants as instrumental variables cannot avoid biases caused by population stratification and compensatory processes during development. As our data were derived solely from patients of European ancestry, this may not be representative of other populations. Future studies should employ diverse datasets to explore the causal relationship between type 2 diabetes and CRAO more broadly. Second, the lower power value in the validation dataset likely resulted from its smaller sample size. Future efforts should focus on acquiring larger datasets to enhance statistical robustness and more effectively validate the relationship between type 2 diabetes and CRAO. Third, although we have adjusted for common risk factors for CRAO in our analysis, the influence of other unexplored factors cannot be completely ruled out. Our team is actively collecting case data from CRAO patients. Upcoming research will utilize longitudinal studies and genomics to address the current limitations of our study.

## 5 Conclusions

In summary, our result show that genetically predicted type 2 diabetes is causally associated with CRAO in two datasets based on patients of European ancestry. This suggests that prevention and management of type 2 diabetes may have the potential to mitigate the risk of CRAO.

## Data availability statement

The original contributions presented in the study are included in the article/supplementary material. Further inquiries can be directed to the corresponding author.

## Ethics statement

Ethical review and approval was not required for the study on human participants in accordance with the local legislation and institutional requirements. Written informed consent from the patients/participants or patients/participants legal guardian/next of kin was not required to participate in this study in accordance with the national legislation and the institutional requirements.

## Author contributions

TL: Writing – review & editing, Writing – original draft, Software, Project administration, Methodology, Investigation, Formal analysis, Data curation. SW: Writing – review & editing, Validation, Supervision. QL: Writing – review & editing, Validation, Supervision. ZL: Writing – review & editing, Validation. XML: Writing – review & editing, Supervision. LP: Writing – review & editing, Validation. XPL: Writing – review & editing, Validation. WG: Writing – review & editing, Validation. PL: Writing – review & editing, Validation. NZ: Writing – review & editing, Validation.
