# Supplementary material for: Causal association of type 2 diabetes with central retinal artery occlusion: a Mendelian randomization study
Source: Front Endocrinol (Lausanne). 2024 Aug 8;15:1379549. doi: 10.3389/fendo.2024.1379549 (PMC11338930; doi:10.3389/fendo.2024.1379549)
Supplement: Supplementary file 1 [file DataSheet_1.doc]

**Supplementary Datasets 1**. Summary statistics for each SNP used as instruments for type 2 diabetes from ebi-a-GCST007515

| No. | SNP | Chr | Position | EA | OA | eaf | beta | se | P |
| --- | --- | --- | --- | --- | --- | --- | --- | --- | --- |
| 1 | rs2296172 | 1 | 39835817 | G | A | 0.1929 | 0.0602 | 0.0077 | 6.73E-16 |
| 2 | rs340874 | 1 | 214159256 | C | T | 0.5032 | 0.0454 | 0.0061 | 1.41E-14 |
| 3 | rs35720761 | 2 | 43519977 | T | C | 0.105 | -0.0733 | 0.0101 | 4.58E-15 |
| 4 | rs13389219 | 2 | 165528876 | T | C | 0.3778 | -0.0647 | 0.0068 | 2.75E-24 |
| 5 | rs7572857 | 2 | 65296798 | A | G | 0.1541 | -0.0524 | 0.009 | 8.25E-09 |
| 6 | rs243021 | 2 | 60584819 | A | G | 0.479 | 0.0457 | 0.0063 | 4.65E-14 |
| 7 | rs2943641 | 2 | 227093745 | C | T | 0.6718 | 0.0588 | 0.0067 | 1.87E-18 |
| 8 | rs1260326 | 2 | 27730940 | C | T | 0.6296 | 0.0609 | 0.0063 | 5.31E-25 |
| 9 | rs1801282 | 3 | 12393125 | G | C | 0.1128 | -0.0848 | 0.0108 | 1.36E-17 |
| 10 | rs11708067 | 3 | 123065778 | G | A | 0.2055 | -0.0726 | 0.0076 | 3.56E-24 |
| 11 | rs7633675 | 3 | 185510613 | G | T | 0.3476 | 0.0967 | 0.0064 | 4.26E-55 |
| 12 | rs4607103 | 3 | 64711904 | T | C | 0.2647 | -0.0399 | 0.0071 | 9.35E-09 |
| 13 | rs1801212 | 4 | 6302519 | A | G | 0.7481 | 0.0683 | 0.0074 | 1.10E-24 |
| 14 | rs6813195 | 4 | 153520475 | T | C | 0.3102 | -0.0485 | 0.0069 | 1.10E-13 |
| 15 | rs459193 | 5 | 55806751 | G | A | 0.6992 | 0.0529 | 0.0069 | 2.82E-15 |
| 16 | rs2307111 | 5 | 75003678 | C | T | 0.4379 | -0.0528 | 0.007 | 1.57E-15 |
| 17 | rs35658696 | 5 | 102338811 | G | A | 0.045 | 0.1251 | 0.0166 | 1.18E-16 |
| 18 | rs4457053 | 5 | 76424949 | A | G | 0.7298 | -0.0402 | 0.0073 | 8.81E-11 |
| 19 | rs9388489 | 6 | 126698719 | G | A | 0.5114 | 0.0337 | 0.0065 | 1.60E-08 |
| 20 | rs9379084 | 6 | 7231843 | A | G | 0.1157 | -0.0795 | 0.0121 | 1.14E-13 |
| 21 | rs2395163 | 6 | 32387809 | C | T | 0.2004 | 0.0519 | 0.0076 | 1.34E-11 |
| 22 | rs7756992 | 6 | 20679709 | G | A | 0.3164 | 0.1025 | 0.0065 | 1.86E-59 |
| 23 | rs1077394 | 6 | 31610384 | T | C | 0.6509 | 0.0399 | 0.0066 | 7.64E-10 |
| 24 | rs2206277 | 6 | 50798526 | T | C | 0.1946 | 0.0474 | 0.008 | 4.31E-10 |
| 25 | rs864745 | 7 | 28180556 | C | T | 0.4569 | -0.0747 | 0.0064 | 1.17E-34 |
| 26 | rs2191349 | 7 | 15064309 | T | G | 0.5498 | 0.0488 | 0.006 | 1.17E-18 |
| 27 | rs730497 | 7 | 44223721 | A | G | 0.1624 | 0.0476 | 0.0081 | 4.01E-10 |
| 28 | rs972283 | 7 | 130466854 | G | A | 0.5604 | 0.0435 | 0.0067 | 1.66E-11 |
| 29 | rs13266634 | 8 | 118184783 | T | C | 0.3093 | -0.0904 | 0.0068 | 1.85E-47 |
| 30 | rs516946 | 8 | 41519248 | C | T | 0.7785 | 0.0655 | 0.0074 | 1.87E-20 |
| 31 | rs328 | 8 | 19819724 | G | C | 0.0966 | -0.0524 | 0.0102 | 6.82E-09 |
| 32 | rs10965250 | 9 | 22133284 | A | G | 0.1795 | -0.1258 | 0.0089 | 9.73E-47 |
| 33 | rs2796441 | 9 | 84308948 | A | G | 0.4107 | -0.0379 | 0.0064 | 1.08E-09 |
| 34 | rs60980157 | 9 | 139235415 | T | C | 0.2286 | -0.062 | 0.008 | 3.19E-16 |
| 35 | rs10758593 | 9 | 4292083 | A | G | 0.4235 | 0.0479 | 0.0061 | 1.95E-16 |
| 36 | rs505922 | 9 | 136149229 | C | T | 0.371 | 0.0376 | 0.0065 | 9.07E-10 |
| 37 | rs7903146 | 10 | 114758349 | T | C | 0.2665 | 0.2325 | 0.0067 | 1.00E-200 |
| 38 | rs10906115 | 10 | 12314997 | G | A | 0.3971 | -0.0341 | 0.0061 | 1.28E-08 |
| 39 | rs12571751 | 10 | 80942631 | G | A | 0.4614 | -0.0541 | 0.0061 | 3.39E-21 |
| 40 | rs5015480 | 10 | 94465559 | T | C | 0.4577 | -0.0705 | 0.0063 | 1.57E-30 |
| 41 | rs2237895 | 11 | 2857194 | C | A | 0.4068 | 0.0758 | 0.0069 | 1.96E-29 |
| 42 | rs11603334 | 11 | 72432985 | A | G | 0.149 | -0.0717 | 0.0089 | 9.50E-18 |
| 43 | rs10830963 | 11 | 92708710 | G | C | 0.2849 | 0.0849 | 0.007 | 1.35E-32 |
| 44 | rs5219 | 11 | 17409572 | C | T | 0.6363 | -0.0584 | 0.0063 | 5.68E-22 |
| 45 | rs10842994 | 12 | 27965150 | T | C | 0.1784 | -0.0629 | 0.0079 | 9.43E-16 |
| 46 | rs1531343 | 12 | 66174894 | C | G | 0.1263 | 0.0657 | 0.0095 | 1.31E-13 |
| 47 | rs3764002 | 12 | 108618630 | T | C | 0.2815 | -0.0319 | 0.0069 | 3.33E-08 |
| 48 | rs55834942 | 12 | 121437114 | A | G | 0.1702 | -0.0578 | 0.0083 | 5.45E-13 |
| 49 | rs1359790 | 13 | 80717156 | A | G | 0.2678 | -0.0593 | 0.0071 | 2.90E-17 |
| 50 | rs10146997 | 14 | 79945162 | G | A | 0.2248 | 0.047 | 0.0078 | 1.84E-10 |
| 51 | rs7177055 | 15 | 77832762 | A | G | 0.6575 | 0.0539 | 0.007 | 7.54E-15 |
| 52 | rs4502156 | 15 | 62383155 | C | T | 0.4759 | -0.036 | 0.0063 | 1.20E-09 |
| 53 | rs8042680 | 15 | 91521337 | A | C | 0.4335 | 0.0439 | 0.0067 | 2.32E-11 |
| 54 | rs7202877 | 16 | 75247245 | G | T | 0.1111 | -0.064 | 0.0097 | 5.63E-12 |
| 55 | rs2925979 | 16 | 81534790 | C | T | 0.6949 | -0.0375 | 0.0069 | 2.49E-08 |
| 56 | rs1558902 | 16 | 53803574 | A | T | 0.3732 | 0.098 | 0.0065 | 2.45E-47 |
| 57 | rs7501939 | 17 | 36101156 | C | T | 0.6221 | -0.0607 | 0.0062 | 3.40E-24 |
| 58 | rs781831 | 17 | 3947644 | C | T | 0.4219 | 0.0413 | 0.0062 | 8.26E-11 |
| 59 | rs12602912 | 17 | 65870073 | T | C | 0.2376 | 0.0426 | 0.0078 | 4.74E-09 |
| 60 | rs17782313 | 18 | 57851097 | C | T | 0.2463 | 0.0442 | 0.007 | 3.50E-10 |
| 61 | rs731839 | 19 | 33899065 | A | G | 0.6351 | -0.0379 | 0.0066 | 5.15E-10 |
| 62 | rs8108269 | 19 | 46158513 | G | T | 0.3258 | 0.0562 | 0.007 | 8.26E-17 |
| 63 | rs58542926 | 19 | 19379549 | T | C | 0.0755 | 0.0715 | 0.0114 | 4.77E-12 |
| 64 | rs769449 | 19 | 45410002 | A | G | 0.1127 | -0.0644 | 0.0102 | 7.56E-13 |
| 65 | rs4812831 | 20 | 43018260 | A | G | 0.1307 | 0.0617 | 0.0096 | 8.48E-10 |
| 66 | rs738409 | 22 | 44324727 | G | C | 0.2388 | 0.0396 | 0.0071 | 2.12E-10 |
| 67 | rs41278853 | 22 | 30416527 | G | A | 0.0738 | -0.0826 | 0.0121 | 5.61E-13 |

SNP, single nucleotide polymorphism; EA, effect allele; OA, other allele; eaf, effect allele frequency; se, standard error.
