# Supplementary material for: Causal association of type 2 diabetes with central retinal artery occlusion: a Mendelian randomization study
Source: Front Endocrinol (Lausanne). 2024 Aug 8;15:1379549. doi: 10.3389/fendo.2024.1379549 (PMC11338930; doi:10.3389/fendo.2024.1379549)
Supplement: Supplementary file 2 [file DataSheet_2.doc]

**Supplementary Datasets 2**. Summary statistics for each SNP used as instruments for type 2 diabetes from finn-b-T2D

| No. | SNP | Chr | Position | EA | OA | eaf | beta | se | P |
| --- | --- | --- | --- | --- | --- | --- | --- | --- | --- |
| 1 | rs2943656 | 2 | 227121918 | G | A | 0.6173 | 0.0756 | 0.0111 | 1.03E-11 |
| 2 | rs62137406 | 2 | 43480221 | T | C | 0.04885 | 0.1396 | 0.0249 | 2.04E-08 |
| 3 | rs112694524 | 2 | 43453721 | A | G | 0.0335 | -0.1809 | 0.0306 | 3.47E-09 |
| 4 | rs10184004 | 2 | 165508389 | T | C | 0.3577 | -0.0703 | 0.0113 | 5.17E-10 |
| 5 | rs6786846 | 3 | 170629884 | A | G | 0.681 | 0.0735 | 0.0116 | 2.47E-10 |
| 6 | rs11712037 | 3 | 12344730 | G | C | 0.1708 | -0.1102 | 0.0144 | 2.00E-14 |
| 7 | rs71330995 | 3 | 123124513 | A | G | 0.1878 | -0.0919 | 0.0139 | 3.73E-11 |
| 8 | rs6780171 | 3 | 185503456 | A | T | 0.3063 | 0.0937 | 0.0117 | 1.10E-15 |
| 9 | rs3887925 | 3 | 186665645 | T | C | 0.4629 | 0.0607 | 0.0109 | 2.39E-08 |
| 10 | rs10938397 | 4 | 45182527 | G | A | 0.4735 | 0.0741 | 0.0108 | 7.51E-12 |
| 11 | rs1046317 | 4 | 6304242 | C | T | 0.6106 | 0.0833 | 0.0111 | 6.90E-14 |
| 12 | rs76177300 | 5 | 102143311 | A | G | 0.05776 | 0.1373 | 0.0232 | 3.09E-09 |
| 13 | rs7451008 | 6 | 20673880 | C | T | 0.3291 | 0.1281 | 0.0115 | 5.43E-29 |
| 14 | rs2781655 | 6 | 131881146 | T | C | 0.2218 | 0.0759 | 0.013 | 5.89E-09 |
| 15 | rs9505086 | 6 | 7232186 | C | T | 0.4438 | 0.0642 | 0.0109 | 4.37E-09 |
| 16 | rs878521 | 7 | 44255643 | A | G | 0.2072 | 0.0896 | 0.0133 | 1.82E-11 |
| 17 | rs62492368 | 7 | 150537635 | A | G | 0.3401 | 0.0771 | 0.0114 | 1.56E-11 |
| 18 | rs77655131 | 7 | 102086552 | T | C | 0.1834 | 0.099 | 0.014 | 1.65E-12 |
| 19 | rs10245867 | 7 | 28142186 | T | G | 0.3308 | 0.0671 | 0.0115 | 5.19E-09 |
| 20 | rs11558471 | 8 | 118185733 | G | A | 0.3785 | -0.0816 | 0.0111 | 2.48E-13 |
| 21 | rs28642213 | 9 | 139248082 | G | A | 0.6971 | 0.1012 | 0.0118 | 8.39E-18 |
| 22 | rs10965246 | 9 | 22132698 | C | T | 0.152 | -0.126 | 0.0151 | 8.78E-17 |
| 23 | rs7018475 | 9 | 22137685 | G | T | 0.2788 | 0.1144 | 0.0121 | 2.84E-21 |
| 24 | rs34872471 | 10 | 114754071 | C | T | 0.2011 | 0.3058 | 0.0137 | 6.00E-110 |
| 25 | rs11257658 | 10 | 12309268 | A | G | 0.2647 | 0.086 | 0.0123 | 3.00E-12 |
| 26 | rs10882099 | 10 | 94460650 | C | T | 0.4771 | -0.0789 | 0.0108 | 3.16E-13 |
| 27 | rs114322470 | 10 | 114736670 | G | T | 0.02364 | -0.2192 | 0.0359 | 1.05E-09 |
| 28 | rs182788819 | 10 | 71449878 | T | C | 0.03872 | 0.1553 | 0.028 | 3.05E-08 |
| 29 | rs5215 | 11 | 17408630 | T | C | 0.5286 | -0.0616 | 0.0108 | 1.27E-08 |
| 30 | rs10770143 | 11 | 2195267 | T | C | 0.6621 | -0.0659 | 0.0114 | 8.42E-09 |
| 31 | rs73541184 | 11 | 72428148 | A | G | 0.3086 | -0.0864 | 0.0117 | 1.89E-13 |
| 32 | rs2237897 | 11 | 2858546 | T | C | 0.08139 | -0.1977 | 0.0201 | 8.16E-23 |
| 33 | rs10830963 | 11 | 92708710 | G | C | 0.3567 | 0.1317 | 0.0113 | 1.99E-31 |
| 34 | rs78470967 | 12 | 4521511 | A | T | 0.03951 | -0.2434 | 0.0285 | 1.23E-17 |
| 35 | rs73113806 | 12 | 66179054 | T | C | 0.02329 | 0.255 | 0.0359 | 1.16E-12 |
| 36 | rs74862545 | 12 | 4365572 | T | C | 0.01954 | -0.2575 | 0.0404 | 1.92E-10 |
| 37 | rs112108223 | 12 | 4271088 | A | G | 0.02244 | -0.3726 | 0.038 | 1.04E-22 |
| 38 | rs76895963 | 12 | 4384844 | G | T | 0.03126 | -0.5078 | 0.0339 | 8.71E-51 |
| 39 | rs56348580 | 12 | 121432117 | C | G | 0.2829 | -0.0782 | 0.0121 | 9.50E-11 |
| 40 | rs1397566 | 12 | 71526677 | G | A | 0.4276 | -0.0596 | 0.0109 | 4.57E-08 |
| 41 | rs7998259 | 13 | 80718654 | A | G | 0.3895 | -0.0767 | 0.0112 | 6.18E-12 |
| 42 | rs28553330 | 15 | 62291191 | C | T | 0.0605 | -0.1278 | 0.0229 | 2.44E-08 |
| 43 | rs12449219 | 16 | 77261943 | G | C | 0.05832 | 0.1308 | 0.0232 | 1.76E-08 |
| 44 | rs9940128 | 16 | 53800754 | A | G | 0.4288 | 0.1173 | 0.0109 | 6.11E-27 |
| 45 | rs55993634 | 16 | 75236763 | G | C | 0.08715 | -0.1638 | 0.0194 | 2.95E-17 |
| 46 | rs11263763 | 17 | 36103565 | A | G | 0.646 | -0.0669 | 0.0113 | 3.55E-09 |
| 47 | rs7224685 | 17 | 4014384 | T | G | 0.3016 | 0.0642 | 0.0117 | 4.48E-08 |
| 48 | rs12967878 | 18 | 57826570 | C | T | 0.1809 | 0.0771 | 0.014 | 3.82E-08 |
| 49 | rs2303700 | 19 | 7976529 | C | T | 0.6741 | -0.0672 | 0.0116 | 6.60E-09 |
| 50 | rs7507893 | 19 | 46157860 | A | G | 0.4965 | -0.061 | 0.0109 | 2.02E-08 |
| 51 | rs8100204 | 19 | 19393714 | A | G | 0.1597 | 0.0964 | 0.0149 | 9.42E-11 |
| 52 | rs429358 | 19 | 45411941 | C | T | 0.1827 | -0.0807 | 0.0142 | 1.35E-08 |
| 53 | rs45551238 | 20 | 57607363 | T | C | 0.05006 | -0.2275 | 0.0253 | 2.21E-19 |
| 54 | rs6073386 | 20 | 42888082 | G | A | 0.0366 | 0.186 | 0.0285 | 6.99E-11 |
| 55 | rs8353 | 22 | 20796117 | T | G | 0.3009 | -0.0745 | 0.0118 | 2.71E-10 |

SNP, single nucleotide polymorphism; EA, effect allele; OA, other allele; eaf, effect allele frequency; se, standard error.
