# Supplementary material for: Causal association of type 2 diabetes with central retinal artery occlusion: a Mendelian randomization study
Source: Front Endocrinol (Lausanne). 2024 Aug 8;15:1379549. doi: 10.3389/fendo.2024.1379549 (PMC11338930; doi:10.3389/fendo.2024.1379549)
Supplement: Supplementary file 5 [file DataSheet_5.pdf]

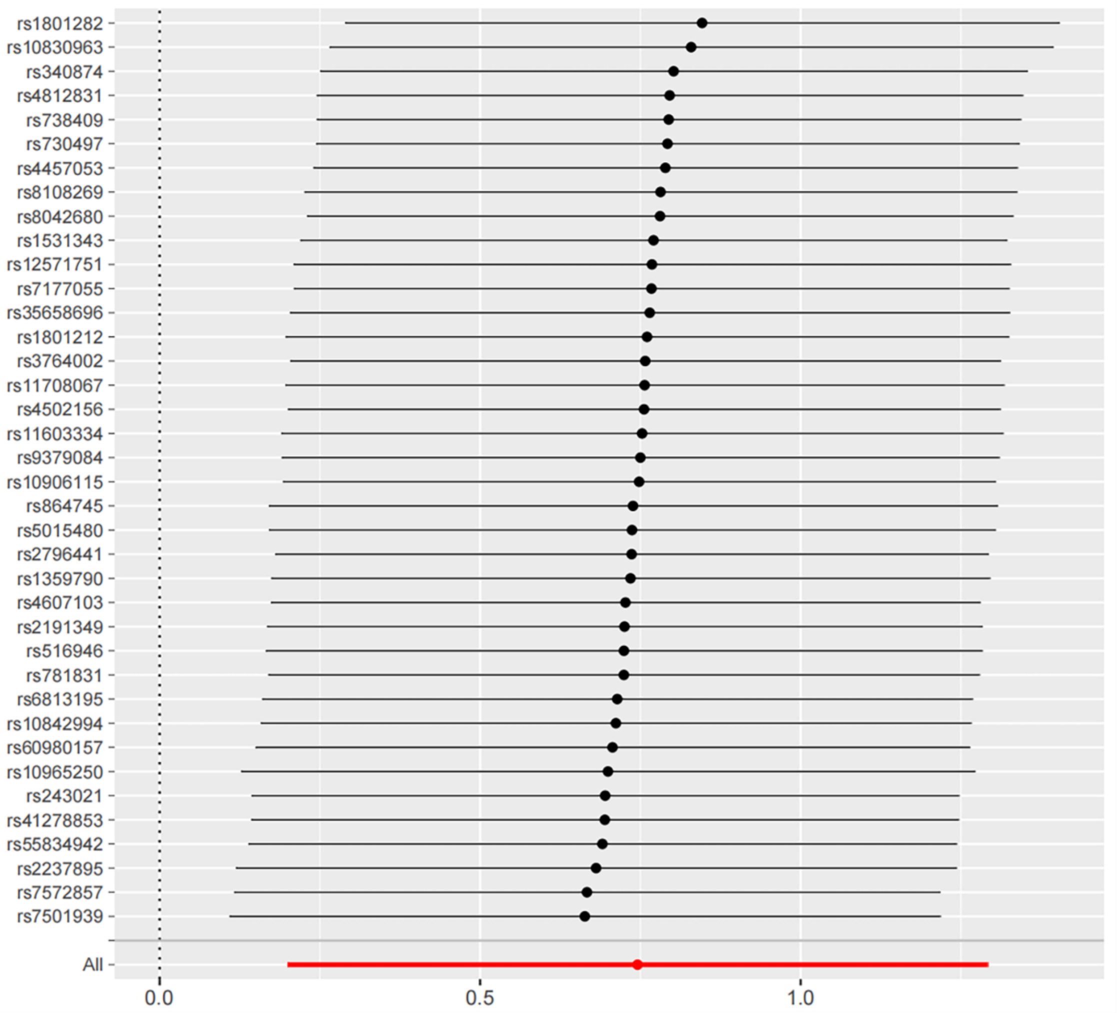

Supplementary figure 1 Leave-one-out analysis plot for type 2 diabetes on the risk of CRAO from the dataset of ebi-a-GCST007515

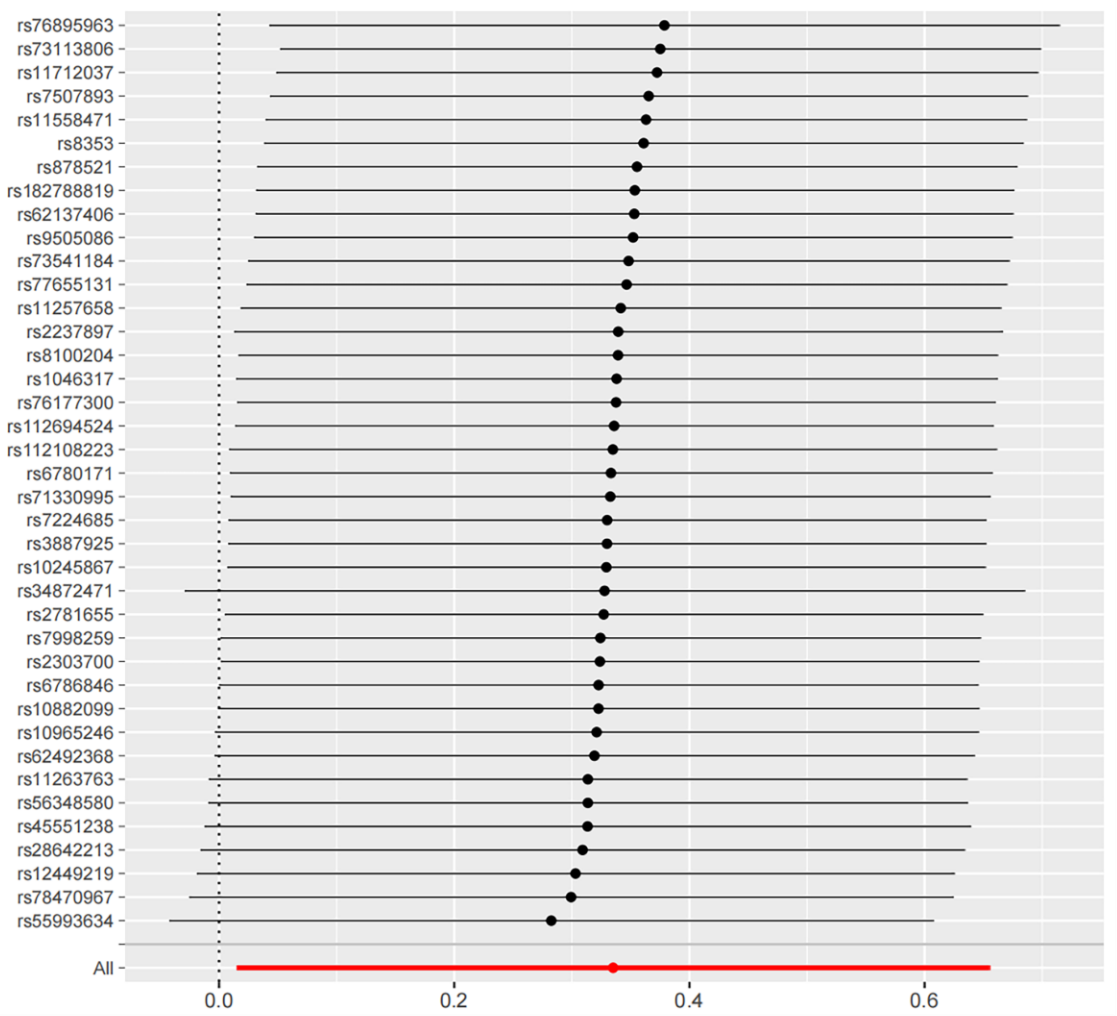

Supplementary figure 2 Leave-one-out analysis plot for type 2 diabetes on the risk of CRAO from the dataset of finn-b-T2D
